# Anxiety in breathing: the impact of air pollution on the mental health of adolescents

**DOI:** 10.3389/fpubh.2025.1552198

**Published:** 2025-05-15

**Authors:** Jiamin Ge, Qi Zhang

**Affiliations:** School of Economics, Anhui University, Hefei, China

**Keywords:** air pollution, mental health, adolescent, China, chronic diseases

## Abstract

**Introduction:**

Nearly all global populations (99%) are exposed to air quality levels that exceed the guidelines set by the World Health Organization, posing significant health risks. Concurrently, mental health issues among adolescents are prevalent. However, existing research on the impact of air pollution on mental health has predominantly focused on older age groups, with limited attention to adolescents.

**Methods:**

This study explores the relationship between air pollution and adolescent mental health by utilizing data from the China Family Panel Studies and county-level air pollution statistics. To address potential endogeneity issues, this paper employs the two-stage least squares (2SLS) method for estimations.

**Results:**

The research reveals a negative impact of air pollution on adolescent mental well-being. Specifically, a one-unit increase in Particulate Matter 2.5 corresponds to a 0.319-point increase in the mental health score, based on an 8-item scale designed to rapidly assess depressive symptoms; higher scores indicate more severe depressive tendencies.

**Discussion:**

Mechanism discussions show that air pollution adversely affects adolescent mental health by negatively influencing height and weight, increasing the risk of chronic diseases, extending computer use time, reducing the likelihood of walking to school, and disrupting sleep quality. Furthermore, the findings indicate that the impact of air pollution is more pronounced among females, urban students, and those below high school level.

## 1 Introduction

The rapid economic and societal advancements since the 21st century have improved human living standards but have also led to significant environmental challenges, particularly in air quality, due to ongoing industrialization and urban expansion. Globally, from 2000 to 2019, 65% of cities experienced an increase in PM_2.5_ levels, contributing to a rising global burden of disease. Approximately seven million deaths annually are attributed to air pollution, affecting nine out of ten people worldwide. Consequently, air pollution has emerged as a pressing global concern.

Scientific research has underscored the detrimental impacts of air pollution on both the environment and human health (1–4). It directly affects the respiratory system, leading to respiratory infections, asthma, and chronic obstructive pulmonary disease (5). Moreover, prolonged exposure is linked to cardiovascular diseases, including heart disease, stroke, and hypertension (6). Studies have also associated air pollution with obesity (7), highlighting its multifaceted health risks. Beyond physical health, air pollution detrimentally affects mental well-being, contributing to anxiety, depression, and increased stress. Fine particulate matter and harmful gases like nitrogen dioxide and ozone can adversely affect the nervous system, exacerbating existing Mental health conditions. Thus, maintaining air quality is crucial for safeguarding Mental health and it can contribute to lessening the global economic burden of mental health issues (8).

Specifically focusing on adolescents, they are facing significant mental health challenges, with 1 in 7 globally experiencing disorders (13% of age-specific disease burden) and 14.8% in China exhibiting depressive symptoms. Prolonged exposure to air pollutants like PM_2.5_ exacerbates emotional distress and impairs cognitive and social functioning, potentially triggering academic decline, family strain, and societal instability (9–11).

Considering it, the impact of air pollution on the Mental health of adolescents is investigated by utilizing data from the China Family Panel Studies (CFPS) conducted by Peking University. Mental health data of adolescents aged 9 to 20 is matched with county-level atmospheric pollution data, and thermal inversion is employed as an instrumental variable. Through the application of two-stage least squares (2SLS), the effect of air pollution on adolescent Mental health is estimated. Therefore, there are three key contributions: Firstly, it explores the correlation between air pollution and adolescent Mental health, addressing a gap in current research and enriching the understanding of the impact of air pollution on Mental health. Secondly, while the majority of research exploring the health effects of air pollution have primarily centered on developed nations (12–15), this study provides valuable insights based on data from China, the largest developing country facing serious environmental pollution issues, thus enriching research on developing countries. Thirdly, by focusing on school-aged children in China and considering the student registration management system, which mitigates residential relocation issues, this study offers a novel perspective on studying the impact of air pollution on permanent residents, addressing a common challenge encountered in air pollution research.

The rest of this paper is organized as follows: Section 2 makes a review of pertinent studies on air pollution. Section 3 and 4 primarily introduce the sources and processing methods of the data, along with an explanation of the adopted model. Section 5 provides a detailed report of the empirical results, and the final section is the conclusion of this paper.

## 2 Literature review

Grossman (16) proposed the Health Demand Model, establishing a theoretical framework for investigating health demand issues in applied economics. This model significantly advances empirical research on health demand behavior within economics. Subsequently, Cropper (17) employs health economic models and cost–benefit analysis to systematically examine the economic ramifications of air pollution on individual and societal health. Building upon this foundation, numerous scholars have investigated the causal effects of air pollution on various health outcomes, including increased infant mortality, elevated mortality rates among the older adult (12–14, 18), delayed child development (19), susceptibility to heart attacks, and chronic lung disease effects (20). Xue et al. (21) investigated the effects of indoor heating on winter air pollution, while Ahmed et al. (22) examined the adverse effects of household indoor air pollution on women’s health, focusing on fertility, pregnancy, and infant birth weight. Additionally, Magazzino et al. (23) explored the potential connection between the COVID-19 outbreak and air pollution. Including increased infant mortality, elevated mortality rates among the older adult (12–14, 18), delayed child development (19) and the women’s health including fertility, pregnancy, and infant birth weight (22). Furthermore, the impact of air pollution on susceptibility to heart attacks and chronic lung disease, has been well documented (20). Additionally, Magazzino et al. (23) explored the potential connection between the COVID-19 outbreak and air pollution.

The rapid globalization and advancement of information technology in the 21st century have made Mental health a key concern in societal development. As individuals face increasing life pressures in today’s complex social environment, mental well-being has gained significant attention. In response, many countries and regions are investing more in Mental health initiatives to raise public awareness and improve individual quality of life. Ferreira et al. (24) found a significant negative correlation between air pollution and life satisfaction in Europe. Arceo et al. (25) highlighted the differing costs of avoidance behavior in developed and developing countries. As the world’s most populous developing nation, China faces severe air pollution challenges amid rapid economic growth. Numerous studies have highlighted the negative impacts of air pollution on public health. He et al. (26) showed that a 10 μg/m^3^ reduction in PM_10_ during the Beijing Olympics led to an 8.36% decrease in mortality, particularly among infants and children. Similarly, Zhang et al. (27) found that a 10% reduction in PM_10_ was associated with an 8.36% decline in mortality and exacerbated depressive symptoms, challenging the idea that economic growth automatically improves happiness.

In the realm of mental health, existing literature has progressively unraveled the multifaceted impacts of air pollution on mental health. Notably, Chen et al. (28) employed a two-stage least squares (2SLS) approach to investigate air pollution’s psychological consequences in China, while Chen et al. (29) focused on the older adult, showing that increased air pollution led to significant declines in their mental health. Also, adopted a regression discontinuity (RD) design to examine the winter heating season in northern China, finding that air pollution worsened mental health, whereas Zhang and Zhang (30) combined difference-in-differences (DID) with 2SLS finding that each 1 μg/m^3^ increase in PM_2.5_ raised the depression index by 3.88% and significantly reduced subjective well-being. Beyond conventional PM_2.5_ metrics, Wang and Luo (31) utilized the Air Quality Index (AQI) to demonstrate pollution’s dual burdens on depression indices and healthcare expenditures—each 1-unit increase in the annual AQI index adds 64.229 billion yuan to national healthcare costs for individuals over 45. Mechanistically, Xie et al. (52) confirmed that air pollution affects Mental health both directly and indirectly by reducing outdoor activities and social integration. In addition, Zheng et al. (32) used big data to explore the link between air pollution and emotional expression on social media, shedding light on public sentiment regarding air quality.

In conclusion, while existing research has extensively explored the impacts of air pollution on adults, particularly middle-aged and older adult populations, studies focusing on adolescents are relatively scarce. Most research on adolescents has concentrated on their academic performance, attendance, and related issues (9, 11, 33), with fewer studies examining adolescent Mental health. Adolescents, being at a crucial stage of physical and mental development, are particularly sensitive and adaptive to their environment. As a result, those living in areas with poor air quality may be more vulnerable to mental health problems. This background provides the rationale for the present study, which investigates the impact of air pollution on adolescent Mental health. This study aims to explore the causal relationship between air pollution and adolescent mental health by integrating adolescent mental health data from the China Family Panel Studies (CFPS) with county-level pollution data. The analysis will discuss the endogeneity issues inherent in the study and the mechanisms of action between the two, and consider the impact on different populations. In the end, we also hope to propose effective strategies and recommendations to mitigate the negative effects of air pollution on adolescents. A more profound comprehension of this issue can facilitate the enhancement of adolescent mental health and contribute to the establishment of a cleaner, healthier living environment for them.

## 3 Methodology and data

### 3.1 Model specifications

The empirical methodology to assess the effect of air pollution on adolescent Mental health is elaborated. To address potential endogeneity issues, this paper employs the two-stage least squares (2SLS) method for estimation. The specific model is set as follows:


$$Y_{i\text{ct}}=\beta_{0}+\beta_{1}P_{i\text{ct}}+\gamma W_{i\text{ct}}+\mu_{i}+\sigma_{t}+\varepsilon_{i\text{ct}}$$

$$P_{i\text{ct}}=\alpha_{0}+\alpha_{1}I_{i\text{ct}}+\gamma W_{i\text{ct}}+\mu_{i}+\sigma_{t}+\varepsilon_{i\text{ct}}$$

$Y_{i\text{ct}}$ represents the Mental health of adolescents, where c denotes the county (district) of the surveyed adolescents, and t represents the year. $P_{i\text{ct}}$ is the mean concentration of PM_2.5_ in the county (district) where the surveyed adolescents are located in year t. $I_{i\text{ct}}$ serves as the instrumental variable for $P_{i\text{ct}}$, representing the count of inversion days in the individual’s city of residence. $W_{i\text{ct}}$ are the weather variables in the same area at the same time, including annual precipitation, sunshine duration, relative humidity and relative wind speed. $\mu_{i}$ represents individual fixed effects, $\sigma_{t}$ represents time fixed effects, and $\varepsilon_{i\text{ct}}$ stands for the random error term.

This paper addresses two aspects of endogeneity: (1) Measurement errors in air data: Satellite-based air pollution data, while suitable for large-scale studies, may introduce measurement errors when used at finer spatial/temporal resolutions, particularly for individual-level exposure. Meteorological data from China Meteorological Administration (CMA) monitoring stations, located away from adolescents’ residences, may also introduce errors. (2) Other factors affecting Mental health: Mental health is influenced by various factors, which may lead to omitted variable bias. Additionally, sample selection bias may arise as individuals sensitive to pollution may choose to relocate away from high-pollution areas, potentially distorting health outcome estimates. According to Chen et al. (34), long-term exposure to high-pollution areas may result in relocation, leading to biased estimates.

To address endogeneity, this paper uses thermal inversion as an instrumental variable. Thermal inversion, a meteorological phenomenon, influences pollutant dispersion and is commonly used as an instrument in air pollution studies due to its exogenous nature (7, 19, 29, 30). When confronted with endogenous problems, firstly, OLS assume exogeneity of air pollution, which is violated if unobserved confounders jointly affect pollution levels and mental health. While generalized method of moments (GMM) can also address endogeneity, it requires multiple valid instruments or strong distributional assumptions. With a single strong IV, 2SLS achieves consistency without overcomplicating the model (35). In addition, 2SLS with meteorological IV is widely validated in environmental epidemiology. For instance, Schlenker and Walker (36) used 2SLS to link ozone pollution to hospitalizations, demonstrating its robustness in causal inference. Similarly, Chen et al. (29) employed thermal inversions in a 2SLS framework to estimate pollution’s impact on cognitive performance, confirming methodological validity. Therefore, to address endogeneity, this paper uses thermal inversion as an instrumental variable. Thermal inversion mainly refers to the situation that the atmospheric temperature increases with the increase of altitude, contrary to the usual situation that the atmospheric temperature decreases with the increase of altitude. This is due to the fact that cold air is below the warmer air, which makes it difficult for the warmer air to circulate, and pollutants diffuse slowly. The formation of thermal inversions is typically uncorrelated with individual-level confounders and independent of economic activities (7). So Thermal inversion is consistent with the principles of correlation and exogeneity of instrumental variables and is commonly used as an instrument in air pollution studies (19, 30, 37). In addition, this paper has complemented and demonstrated the choice of IV with the F-statistic values obtained from the first-stage regression and the references of the existing literature in Section 4.1. As shown in Table 1, the 2SLS first-stage F-statistic passes the test for weak instrumental variables (*F* > 10 in our analysis).

**Table 1:** Effect of thermal inversions on PM_2.5_.

Variables	(1)	(1)
	First	First
	PM_2.5_	PM_2.5_
day1	0.0601***	0.101***
	(3.62)	(6.35)
Weather controls	No	Yes
Observations	3,984	3,980
Individual FE	Yes	Yes
Time FE	Yes	Yes
KPF	13.07	40.26

***p < 0.01, **p < 0.05, *p < 0.1.

The study also accounts for sample selection bias by focusing on adolescents, who are less likely to move due to China’s school registration system. Finally, individual fixed effects are included in the regression model to control for intrinsic health factors.

### 3.2 Indicators selected and data source

#### 3.2.1 Mental health

The Mental health data, as well as individual-level control variables, are sourced from the CFPS which is organized by the *Institute of Social Science Survey* at Peking University. Since its inception in 2010, the CFPS has carried out six survey rounds, encompassing 25 regions across China. CFPS is a nationwide social tracking survey program that tracks data at the individual, family, and community levels. It comprehensively reflects changes in China’s society, economy, demographics, education, and health, thereby offering dependable data for academic research and public policy analysis.

Different assessment tools are used for adolescent Mental health measurement in different years: the Kessler Mental Illness Scale (K6) in 2010, the CES-D20 scale in 2012, the CES-D8 scale in 2016, 2018, and 2020. The K6 scale is primarily for adult Mental health research, while the CES-D scale is applicable to both adults and adolescents. To ensure data consistency and comparability, adolescent Mental health data from CFPS 2016, 2018, and 2020 are selected for analysis in this study.

**Figure 1:**
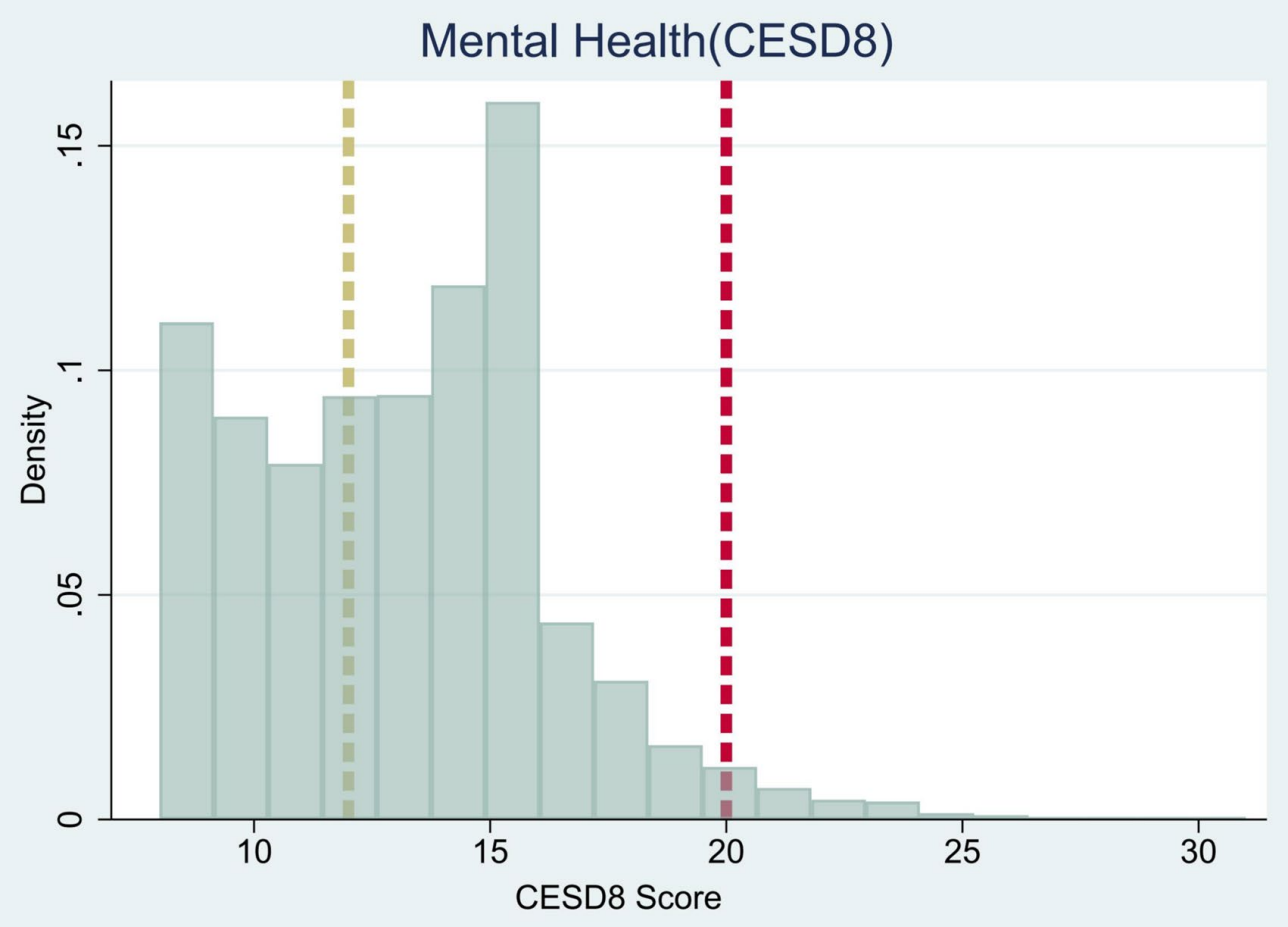
Histogram of the CESD8 score. The figure plots the histogram of the CESD8 score, ranging from 8 to 32. The vertical brown line indicates the cut-off of 12, which is used to define mild depressive symptoms mental illness. The vertical red line indicates the cut-off of 20, which is used to define severe mental illness.

**Figure 2:**
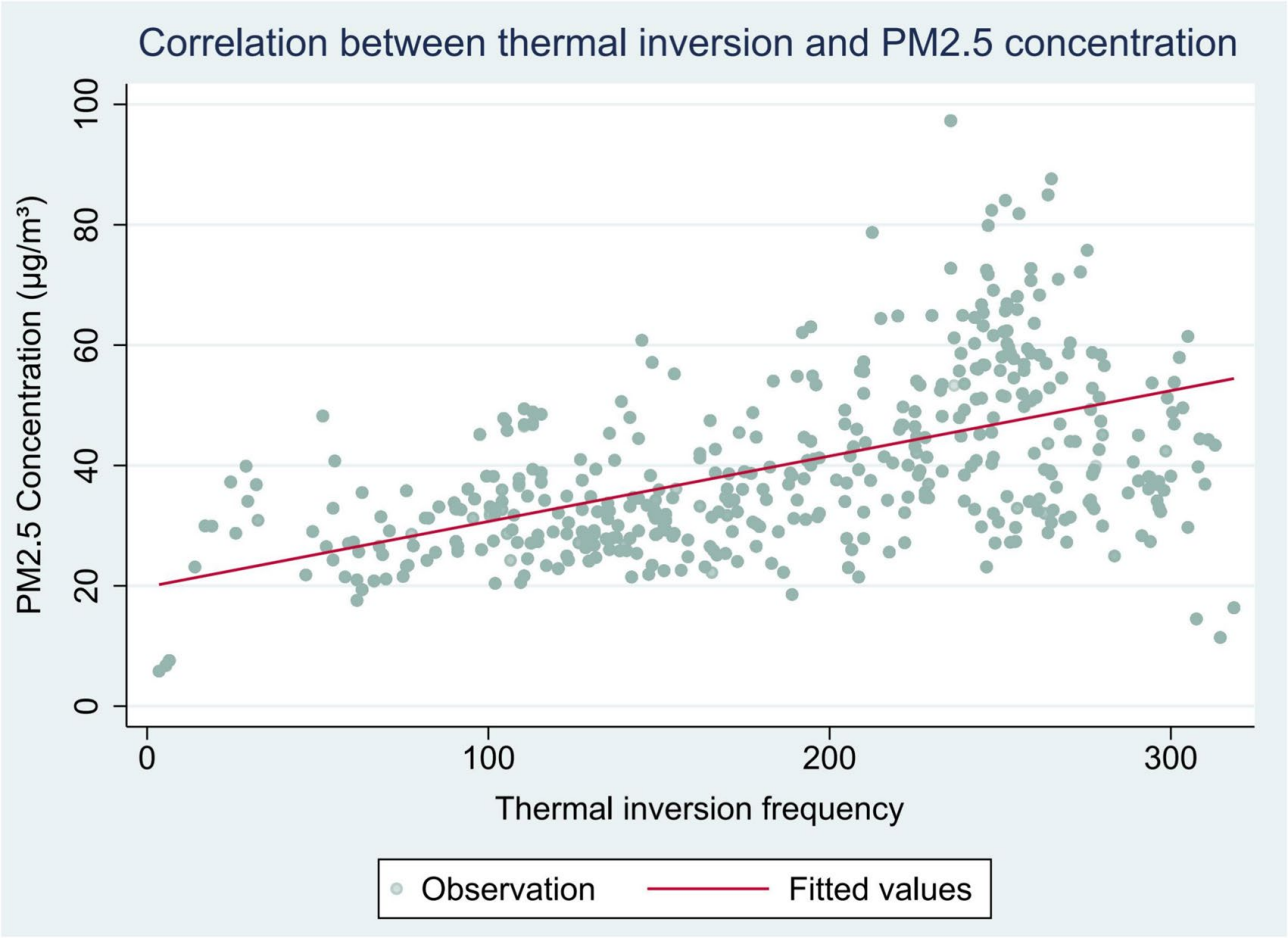
Correlation between thermal inversion and PM2.5 concentration.

The CES-D8 scale, validated across 25 countries in the general population (38), assesses depressive symptoms. In the CFPS questionnaire, children reported the frequency of experiencing eight feelings over the past week: (1) depression, (2) everything feeling like an effort, (3) restless sleep, (4) happiness, (5) loneliness, (6) enjoyment of life, (7) sadness, and (8) difficulty getting going. Respondents rate each feeling on a scale of 1 (none) to 4 (almost every day or every day). Positive items such as happiness and enjoyment of life are reverse coded. The total score ranges from 8 to 32, where higher scores suggest more pronounced depressive symptoms (Figures 1, 2).

#### 3.2.2 Air pollution

The air pollution data are from Washington University in St. Louis. In this study, county-level PM_2.5_ concentration serves as a proxy indicator of air pollution. Most air pollution data in China come from ground-based detection stations provided by the *National Environmental Monitoring Center of the Ministry of Ecology and Environment*. However, due to limited station coverage, there is substantial missing data at the county level. Additionally, Chen et al. (28) emphasize that ground-based monitoring stations are inadequate for studying specific pollutants, such as PM_2.5_, and may be subject to measurement bias due to human interference. Therefore, this study primarily selects PM_2.5_ data from the *Atmospheric Composition Analysis Group* at Washington University, which combines satellite observations from multiple NASA instruments with the GEOS-Chem chemical transport model. Using geographically weighted regression, the data are calibrated based on ground-based observations to generate PM_2.5_ measurements with varying levels of accuracy. The final set of data processed contains PM_2.5_ data from 162 counties in 25 provinces and cities across China.

#### 3.2.3 Thermal inversions

The data of thermal inversions are derived from National Aeronautics and Space Administration (NASA). Thermal inversion, a critical meteorological phenomenon frequently employed as an instrumental variable in studies related to air pollution, is investigated in this paper. Following prior research (7), MERRA-2 data from NASA are acquired and the near-surface layers are extracted. These data are then transformed into district and county panel data. The temperature differential between the first and second layers are utilized to identify inversion days, serving as the instrumental variable *thermal inversion day1*. Furthermore, the temperature difference between the first and third layers is employed to establish a new criterion for thermal inversion, creating a supplementary instrumental variable *day2* for subsequent robustness testing.

#### 3.2.4 Meteorological data

Meteorological data, including annual average relative wind speed, relative humidity, precipitation, and sunshine duration, are collected from various monitoring stations within the China Meteorological Administration (CMA). To link these data with the individual county database of Mental health interviews, a conversion is conducted using an inverse distance weighting algorithm, aligning the meteorological data from the stations to the county level where the youth participants were located for the interviews.

In summary, this paper utilizes a total of 6,943 respondents from the 2016, 2018, and 2020 waves. Individual codes from the CFPS database are used to form unbalanced panel data spanning the same period. Subsequently, all the data is matched with the respondents’ counties at the county level and logarithmized to mitigate the effect of heteroskedasticity. Table 2 presents descriptive analysis of these variables.

**Table 2:** Descriptive statistics.

Variables	(1)	(2)	(3)	(4)	(5)	(6)
	*N*	Mean	p50	SD	Min	Max
cesd8	6,302	13.22	13.22	3.257	8	31
day1	6,916	191.7	191.7	71.54	3.500	318.5
day2	6,916	134.7	134.7	56.90	0	256
PM_2.5_	6,929	40.69	40.69	15.13	5.824	97.30
Wind	6,929	2.186	2.186	0.484	1.066	4.287
Humidity	6,924	68.65	68.65	9.245	46.27	85.35
Rainfall	6,924	996.8	996.8	532.4	220.8	2,697
Sunshine	6,929	2,006	2,006	471.8	935.5	3,063
Age	6,937	14.60	14.60	3.316	9	20
Gender	6,937	0.544	0.544	0.498	0	1
Height	6,594	158.2	158.2	15.00	110	184
Weight	6,550	98.20	98.20	28.87	41	180
lnfincome1_per	6,883	9.518	9.518	0.926	4.828	14.51

## 4 Results and analysis

### 4.1 First-stage results

To in light of the aforementioned considerations, this study employs thermal inversion as an instrumental variable with the objective of elucidating the causal link between air pollution and adolescent Mental health. In accordance with the definition proposed by Arceo et al. (25), thermal inversion is defined as the temperature at the second level (320 m) being higher than the temperature at the first level (110 m). Deschenes et al. (7) conduct a comprehensive analysis of the relationship between thermal inversion and GDP at the county level in China, demonstrating that thermal inversion is not associated with economic activities. Moreover, several studies (7, 25, 27, 28, 39, 52) have employed thermal inversion as an instrumental variable for air pollution, thereby confirming a significant effect of thermal inversion on pollution levels.

Table 1 presents the findings of the first robustness test. Column (1) incorporates individual fixed effects along with year-by-month fixed effects, while column (2) adds weather controls such as annual average relative wind speed, relative humidity, precipitation, and sunshine duration. The results clearly indicate a significantly positive correlation between thermal inversions and PM_2.5_ concentrations. Specifically, data from the second column shows that for each additional thermal inversion event in the preceding 12 months, the PM_2.5_ concentration rises by 0.101 μg/m^3^ during the corresponding period.

### 4.2 Baseline results

Table 3 presents the IV estimate and the OLS estimate regarding the impact of PM_2.5_ on adolescent mental illness. The second-stage regression of the 2SLS analysis indicates that the effect of air pollution on depressive tendencies is statistically significant. In Column (1), the model includes only individual fixed effects and time fixed effects. The results show that a 1 μg/m^3^ increase in PM_2.5_ concentrations is associated with a statistically significant increase of 0.340 units in the CESD8 score, significant at the 5% level. Moving to Column (2), weather controls are added to ensure that air pollution is the primary channel through which thermal inversion affects mental health. Although the estimated effect slightly decreases to 0.319 units, it remains statistically significant at the 1% level. This finding is consistent with related literature based on CFPS data (27, 28, 30), which suggests that adolescent mental well-being is highly sensitive to air pollution. Finally, a comparison between the 2SLS and OLS results reveals that OLS estimates are generally lower than 2SLS estimates. This discrepancy may be due to the fact that satellite-monitored air pollution data are less specific to individual exposure and may thus underestimate the true impact of air pollution on individuals, potentially due to classical measurement errors.

**Table 3:** Effect of air pollution on adolescent mental health.

1	(1)	(2)	(3)	(4)
	IV		OLS	
Variables	cesd8	cesd8	cesd8	cesd8
PM_2.5_	0.340**	0.319***	0.136***	0.118***
	(2.18)	(3.69)	(12.44)	(10.25)
Weather controls	No	Yes	No	Yes
Observations	3,984	3,980	6,287	6,282
Individual FE	Yes	Yes	Yes	Yes
Time FE	Yes	Yes	Yes	Yes
KPF	13.07	40.26		

***p < 0.01, **p < 0.05, *p < 0.1.

### 4.3 Robustness checks

Table 4 presents robustness check results. Column (1) shows two-stage least squares regression with weather controls. Column (2) excludes weather controls, using only PM_2.5_ concentration in the fixed effects regression. Column (3) adds net household income per capita while controlling for weather variables. Column (4) tests robustness by using an alternative method to define thermal inversion, replacing the second layer (320 m) with the third layer (540 m). The last column shows results statistically significant at the 1% level. As in Table 3, the results are consistent with the baseline regression and statistically significant at the 1% level.

**Table 4:** Robustness checks.

Variables	(1)	(2)	(3)	(4)	(5)
	Baseline	No weather	Other controls	New IV	Winsorize
	cesd8	cesd8	cesd8	cesd8	cesd8_w
PM_2.5_	0.319***	0.340**	0.330***	0.321***	0.321***
	(3.69)	(2.18)	(3.55)	(3.10)	(3.74)
lnfincome1_per			0.213		
			(1.00)		
Weather controls	Yes	No	Yes	Yes	Yes
Observations	3,980	3,984	3,959	3,980	3,980
Individual FE	Yes	Yes	Yes	Yes	Yes
Time FE	Yes	Yes	Yes	Yes	Yes
KPF	40.26	13.07	36.10	23.69	40.26

***p < 0.01, **p < 0.05, *p < 0.1.

### 4.4 Heterogeneity analysis

The heterogeneity of the respondents is employed based on their urban and rural household registration, gender, and educational stage (below junior high school and above senior high school). Table 5 reports the specific results.

**Table 5:** Heterogeneity analysis.

Variables	(1)	(2)	(3)	(4)	(5)	(6)
	Rural	Urban	Girl	Boy	Junior	Senior
	cesd8	cesd8	cesd8	cesd8	cesd8	cesd8
PM_2.5_	1.047	0.290**	0.356**	0.275***	0.447***	0.163
	(1.35)	(2.40)	(2.34)	(3.00)	(2.61)	(1.45)
Weather controls	Yes	Yes	Yes	Yes	Yes	Yes
Observations	1,884	1,919	1,769	2,153	2,091	874
Individual FE	Yes	Yes	Yes	Yes	Yes	Yes
Time FE	Yes	Yes	Yes	Yes	Yes	Yes
KPF	1.84	17.05	14.60	33.59	14.30	15.34

***p < 0.01, **p < 0.05, *p < 0.1.

Respondents are categorized into rural and urban groups based on household registration. The results show that air pollution significantly affects the mental well-being of adolescents in urban areas, consistent with studies on adults and older adults (30). This difference may stem from the fact that urban residents tend to place greater importance on air pollution than rural residents.

In columns (3) and (4), the sample is divided by gender. The analysis reveals that air pollution has a more significant impact on the mental well-being of girls, aligning with findings by Zhang et al. (27), who noted that females are more susceptible to the effects of air pollution. One possible explanation is that girls are more emotionally reactive during adolescence, making them more vulnerable to environmental factors. Additionally, studies on child development in India (19) suggest that female adolescents are more prone to growth and developmental delays caused by air pollution. Overall, these findings highlight that girls’ mental health is particularly sensitive to the harmful effects of air pollution, underscoring the need for targeted measures to reduce their exposure.

The sample is further divided into two groups based on educational stage: elementary and junior high school students, and high school students and above. The results indicate higher regression coefficients for the younger group, suggesting that younger students are more vulnerable to the mental health effects of air pollution. This may be because younger students are less physically mature, spend more time outdoors, and have greater exposure to pollution, whereas high school students, who focus more on academics, spend less time outside. Additionally, high school students may have better physical resilience and awareness of environmental protection, making them less susceptible. Zhang et al. (27) also found that families with younger children (under 16 years old) are more vulnerable to the mental health impacts of air pollution.

Columns (1), (2), and (3) of Table 6 report the interaction coefficients for gender × age, gender × education stage, and gender × urban. To mitigate multicollinearity concerns, age was centered at its mean. Results indicate that only the gender-urban interaction is statistically significant and positive, suggesting that urban–rural disparities significantly moderate gender-based sensitivities to air pollution. Specifically, urban male adolescents (gender = 1 and urban = 1) exhibit PM_2.5_ mental health effects 1.546 units higher than rural females (baseline group), likely due to urban males’ greater outdoor activity rates and traffic-related pollution exposure. In contrast, the gender-education and gender-age interactions are statistically insignificant, implying that educational resources and age progression do not amplify gender disparities. These findings highlight that urban–rural contexts, by shaping behavioral patterns and exposure pathways, serve as the primary moderator of gender-specific vulnerabilities, whereas other dimensions of gender heterogeneity require larger samples or longitudinal data for further exploration.

**Table 6:** Heterogeneity analysis.

Variables	(1)	(2)	(3)
	cesd8	cesd8	cesd8
Gender × Centered_age	−0.131		
	(−1.58)		
Gender ×Edu_stage		0.314	
		(0.84)	
Gender × Urban			1.546*
			(1.72)
PM_2.5_	0.459**	0.307***	0.363***
	(2.39)	(3.35)	(3.41)
Observations	3,980	3,625	3,910
Individual FE	Yes	Yes	Yes
Time FE	Yes	Yes	Yes

***p < 0.01, **p < 0.05, *p < 0.1.

## 5 Mechanism discussions

In this section, several aspects of life as indicators for examination are selected, including physical health, chronic disease, electronic device use time, the transportation mode to school, and sleep quality, in order to investigate the mechanism of air pollution’s impact on adolescents’ Mental health.

This paper first selects physiologic indicators that directly measure health status, including height, weight, and chronic disease status. In regard to height, a study by Rees et al. (40) found that height was negatively correlated with depressive symptoms in a group of females between the ages of 17 and 19 and males between the ages of 12 and 19. Conversely, Le et al. (41) found that adolescents with above-average height demonstrated higher levels of subjective well-being as well as lower tendencies to be depressed as compared to a group with below-average height. In terms of body weight, adolescents may experience low self-esteem as a consequence of being overweight or obese (42). This low self-esteem has a further detrimental effect on their Mental health, for instance, by increasing the risk of depression and anxiety. A substantial body of research has consistently demonstrated that air pollution plays a significant role in the development of cardiovascular problems, respiratory and cerebrovascular diseases. Furthermore, these chronic conditions can have a detrimental impact on Mental health (43–45, 53).

In addition to direct health indicators, this paper also explores the potential indirect impact of air pollution on adolescents’ Mental health. The way to school is one of the most important parts of young people’s daily lives. Active commuting to school (ACS), such as walking and cycling, could be a source of physical activity, contributing to the improvement of youth cardiovascular health (46). Active commuters exhibit lower levels of heart rate, blood pressure, and perceived stress (47, 48). Reilly et al. (49) found that adolescents perceived social media as a potential threat to their mental wellbeing. Viner et al. (50) conducted a study to analyze three potential mechanisms by which frequent use of social media may negatively impact the Mental health of adolescents, including an increased risk of cyberbullying, a reduction in the time spent sleeping and engaging in physical activity, and an elevated exposure to harmful content. For this reason, data on computer use time and adolescent sleep quality scores are selected as representative factors to further explore how air pollution indirectly affects adolescents’ Mental health by influencing their daily habits.

The specific results are presented in Table 7. Columns (1) to (2) report the impact of PM_2.5_ on physical health. In column (1) and (2), the results show that air pollution has a significant negative impact on the both height and weight of adolescents. Specifically, a 1 μg/m^3^ increase in PM_2.5_ concentrations decreases the height and weight by 0.790 and 1.293 units, respectively. This finding is consistent with the previous studies (19). The preceding analysis indicates that depressive tendencies were more pronounced in adolescents exhibiting below-average height (41). Furthermore, inadequate growth in height and weight can also impede adolescents’ capacity to withstand the detrimental effects of air pollution.

Column (3) report the effect of PM_2.5_ on chronic disease. The CFPS questionnaire inquired as to whether the respondent had been diagnosed with a chronic disease by a physician in the preceding 6 months. The respondents are assigned a value of 0 for undiagnosed chronic illness and a value of 1 for diagnosed chronic illness. The evidence indicates that air pollution increases the likelihood of developing chronic disease in adolescents. Specifically, a 1 μg/m^3^ increase in PM_2.5_ concentrations is associated with a 0.005-unit increase in the prevalence of chronic disease (Table 7).

**Table 7:** Mechanism tests.

Variables	(1)	(2)	(3)	(4)	(5)	(6)
	Height	Weight	Illness	Sleep quality	Computer	ACS
PM_2.5_	−0.790***	−1.293***	0.002	0.003	0.003	0.305
	(−4.06)	(−3.86)	(0.04)	(0.12)	(0.41)	(0.93)
Observations	4,269	4,214	2,719	3,963	3,970	1,943
Individual FE	Yes	Yes	Yes	Yes	Yes	Yes
Time FE	Yes	Yes	Yes	Yes	Yes	Yes
KPF	34.19	32.26	20.17	13.86	36.47	19.36

***p < 0.01, **p < 0.05, *p < 0.1.

In column (4), the dependent variable is sleeping quality, which is based on the CFPS questionnaire on *I have poor sleep quality* with one for good sleep quality and five for bad. Therefore, a higher score signifies poorer sleep quality. Specifically, for every 1 μg/m^3^ increase in PM_2.5_ concentrations, the poor sleep quality score rises by 0.003 units. Column (5) presents the effect of PM_2.5_ on computer use time. The results indicate that air pollution significantly increases adolescents’ computer use time. Specifically, each 1 μg/m^3^ increase in PM_2.5_ concentration results in an increase in the extent of computer use by adolescents by 0.003 units. According to Viner et al. (50), frequent use of social media can affect teenagers’ sleep and further induce Mental health problems.

With regard to the mode of travel to school utilized by adolescents (column 6), values are assigned based on the CFPS questionnaire on *mode of travel from residence to school*. These values ranged from 1 to 7, with 1 representing walking, 2 representing bicycles or tricycles, 3 representing motorized bicycles or electrically powered tricycles, and 4 representing buses or cars. The findings indicate that air pollution prompts adolescents to opt for alternative modes of transportation to school, rather than walking, which indirectly resulted in a reduction in the amount of exercise they engaged in. Physical activity is recognized as an important lifestyle for mental health promotion (51). The negative health effects of air pollution may contribute to an increase in anxiety and depression among adolescents.

## 6 Conclusion and policy implications

This paper explores the effects of air pollution on the Mental health of adolescents, revealing that a 1 μg/m^3^ increase in PM_2.5_ concentration correlates with a 0.319-point rise in the CES-D8 depression score. This highlights the negative impact of air pollution on their psychological well-being. The heterogeneity analysis shows that urban residents, female adolescents, and those below high school level are more vulnerable to the Mental health impacts of air pollution. The paper further explores the mechanisms, demonstrating that air pollution adversely affects adolescent Mental health through negative impacts on physical development, chronic disease risk, increased computer usage, reduced walking to school, and disrupted sleep.

Based on the profound results gained from the study, it is crucial to propose practical recommendations to alleviate the negative effects of air pollution on adolescent Mental health. At the governmental level, key actions include deploying low-cost PM_2.5_ sensors in areas with high adolescent foot traffic, such as schools and community centers, to establish real-time monitoring networks. These networks can be integrated with AI-driven platforms that issue tiered alerts (e.g., SMS and app notifications) during high-risk periods, such as peak commuting hours and sports activities. Additionally, schools could incorporate air pollution literacy into their health curricula, teaching students about protective strategies like wearing masks and using air purifiers. On high-pollution days, outdoor activities should be replaced with indoor exercise or mindfulness sessions to minimize both exposure and stress. Regular mental health screenings and free counseling services for high-risk students, alongside teacher training to recognize pollution-related symptoms (e.g., anxiety, poor concentration), are crucial in identifying early signs of mental health strain. Furthermore, parents can play an active role by monitoring air quality forecasts, adjusting children’s activity schedules to avoid peak pollution times, and promoting healthy habits such as balanced nutrition, adequate sleep, and regular physical activity. Collectively, these efforts can create a healthier, more sustainable environment for our youth, helping to mitigate the detrimental effects of air pollution on their mental well-being. This study is also subject to certain limitations: (1) the data employed are limited to questionnaire responses, which may be subject to recall bias and subjective evaluation. Additionally, the sample size of adolescents is relatively small. (2) Due to the high correlation between air pollutants, thermal inversion may also impact other air pollutants, including PM_10_, O_3_ and CO (25). Therefore, even if we choose to use thermal inversion as an instrumental variable for PM_2.5_, we cannot accurately determine the effect of PM_2.5_ itself.

## Data Availability

Publicly available datasets were analyzed in this study. This data can be found here: all data resources have explained in the Section 3.2 of the article.
